# Development of a mortality score to assess risk of adverse drug reactions among hospitalized patients with moderate to severe chronic kidney disease

**DOI:** 10.1186/s40360-019-0318-6

**Published:** 2019-07-08

**Authors:** Monica Danial, Mohamed Azmi Hassali, Ong Loke Meng, Yoon Chee Kin, Amer Hayat Khan

**Affiliations:** 1 Discipline of Social and Administrative Pharmacy, School of Pharmaceutical Sciences, Universiti Sains Malaysia, 11800 Minden, Penang, Malaysia; 2 Clinical Research Center (CRC) Hospital Pulau Pinang, Institute For Clinical Research, Ministry of Health Malaysia (MOH), Penang, Malaysia; 3 Discipline of Clinical Pharmacy, School of Pharmaceutical Sciences, Universiti Sains Malaysia, 11800 Minden, Penang, Malaysia; 4 Clinical Research Center (CRC) Hospital Seberang Jaya, Institute For Clinical Research, Ministry of Health Malaysia (MOH), Penang, Malaysia

**Keywords:** Chronic kidney disease (CKD), Adverse events, Mortality risk prediction model, Laboratory variables

## Abstract

**Background:**

Chronic kidney disease (CKD) is a significant health burden that increases the risk of adverse events. Currently, there is no validated models to predict risk of mortality among CKD patients experienced adverse drug reactions (ADRs) during hospitalization. This study aimed to develop a mortality risk prediction model among hospitalized CKD patients whom experienced ADRs.

**Methods:**

Patients data with CKD stages 3–5 admitted at various wards were included in the model development. The data collected included demographic characteristics, comorbid conditions, laboratory tests and types of medicines taken. Sequential series of logistic regression models using mortality as the dependent variable were developed. Bootstrapping method was used to evaluate the model’s internal validation. Variables odd ratio (OR) of the best model were used to calculate the predictive capacity of the risk scores using the area under the curve (AUC).

**Results:**

The best prediction model included comorbidities heart disease, dyslipidaemia and electrolyte imbalance; psychotic agents; creatinine kinase; number of total medication use; and conservative management (Hosmer and Lemeshow test =0.643). Model performance was relatively modest (R square = 0.399) and AUC which determines the risk score’s ability to predict mortality associated with ADRs was 0.789 (95% CI, 0.700–0.878). Creatinine kinase, followed by psychotic agents and electrolyte disorder, was most strongly associated with mortality after ADRs during hospitalization. This model correctly predicts 71.4% of all mortality pertaining to ADRs (sensitivity) and with specificity of 77.3%.

**Conclusion:**

Mortality prediction model among hospitalized stages 3 to 5 CKD patients experienced ADR was developed in this study. This prediction model adds new knowledge to the healthcare system despite its modest performance coupled with its high sensitivity and specificity. This tool is clinically useful and effective in identifying potential CKD patients at high risk of ADR-related mortality during hospitalization using routinely performed clinical data.

## Background

Chronic kidney disease (CKD) is a major health problem for most Southeast Asia (SEA) underdeveloped countries. The exact true prevalence and incidence in the SEA region is not known [1]. CKD prevalence in West Malaysia was estimated at 9.07% [2]. The annual mortality rate caused by CKD increased from 1990 to 2015 at an average rate of 3.4% per year and contributes to 31.4% of the global burden of disability-related years of life, rising at a rate of 1% per year [3–5]. CKD’s mortality rate is estimated at 14 per 100,000 people by 2030 [6].

Adverse drug reactions (ADRs) are defined as drug reactions that are harmful and unintended at normal doses [7]. ADRs are estimated to occur in nearly 20% of drugs that are consumed in the population. It is expected that this number are doubled among hospitalized patients [8]. In Europe, 11.9% of ADRs occurrence rate during hospitalization was reported [9]. Generally, there is still a lack of data on the prevalence of ADRs from adults in SEA countries [10].

The Malaysian Adverse Drug Reactions Advisory Committee (MADRAC), a division of the National Pharmaceutical Regulatory Agency (NPRA), regulates pharmacovigilance activities in Malaysia. ADRs are reported voluntarily by medical professionals, allied health care professionals, drug companies and consumers [11]. ADRs reports are usually submitted via online or manual submission to the NPRA secretariat. NPRA receives annual reports of approximately 9000 to 11,500 ADRs, corresponding to approximately 300 to 387 reports per million people per year [11, 12]. In 10 years, the number of reports had averaged at less than 6000 [13]. Malaysia’s ADR reports have consistently achieved an average completeness score of approximately 0.45 over the past 5 years, increasing gradually to 0.63 in 2014 and 0.72 in 2015 [13]. The incidence of ADRs in paediatric patients in Malaysia in 2012 was 16.8%. However, data on the prevalence of ADRs in Malaysia among the adult population are not available [14].

Each prescribed medication carries its own risk of ADRs, which spans from cosmetic to severe morbidity and mortality due to patient specific reasons [15]. Pre-existing comorbidities can increase the susceptibility of an individual to an idiosyncratic reaction in addition to drugs that can alter cytochrome P450 metabolic pathways resulting in either increased production or impairment of toxic metabolites clearance [16, 17].

The use of predictive risk scores is an emerging approach to reduce adverse drug events associated with admission to hospitals [18]. Clinical risk prediction models include multiple variables to predict the risk of adverse events for a patient [19, 20]. Risk prediction models are accessible in real-world clinical practice. Furthermore, routinely measured laboratory variables would facilitate timely calculation of mortality risk from electronic medical records or laboratory information systems [21].

Therefore, the aim of this study is to develop to a mortality risk score of ADRs among multi-wards hospitalized stages 3 to 5 CKD patients by assessing the risk of mortality in this specific sub-population. The risk score for mortality has significant implications for clinical practice and research. The mortality risk model would facilitate the management of low-risk and high-risk patients according to the levels required to reduce mortality outcomes and further improve the cost-effectiveness of CKD therapy, thereby enhancing the quality of care of CKD patients.

## Methods

### Study setting

This retrospective observational study was conducted in Hospital Pulau Pinang, Malaysia a 1090-beds, second largest General Hospital in Malaysia. Additionally, highest number of ADRs were reported from Hospital Pulau Pinang from 2014 till 2016 [11, 13] which corresponds to the duration of this study. Some of the methods outlined in this manuscript overlap with our earlier published research article [22].

### Study design and data collection

**Fig. 1:**
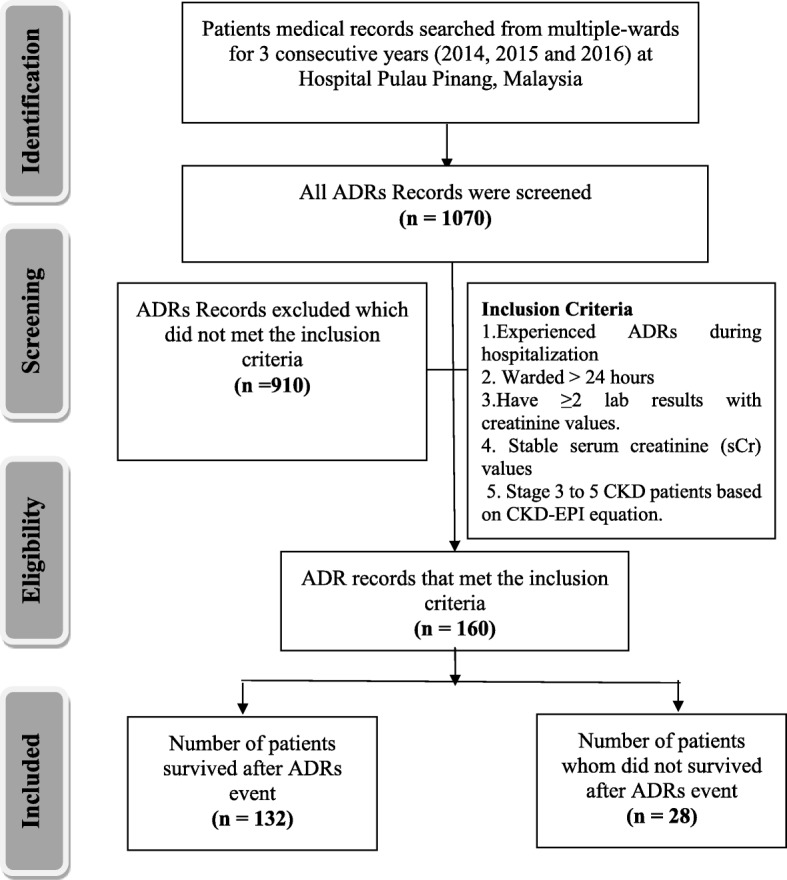
Study flow diagram of identification of chronic kidney disease (CKD) patients’ medical records whom experienced adverse drug reactions (ADRs) during hospitalization for the duration of 2014 till 2016 in Hospital Pulau Pinang, Malaysia

Retrospective data from medical records of patients with CKD stages 3–5 (estimated GFR < 60 ml/min/1.73m^2^) admitted to different wards between 1 January 2014 and 31 December 2016 and experienced ADRs were screened for the purposes of this study (Fig. 1). Data were collected retrospectively from the medical records using a standardized form for each patient. Information gathered included (a) demographic characteristics (b) results of physical examination (c) existing comorbid conditions (d) laboratory tests results (e) medication types (f) date start and end of ADRs and (g) information of ADRs causative drugs [22].

The characterization of the ADR events was done using a 3-step identification method (trigger list/ doctor’s order, an independent reviewer confirmation and an experienced pharmacist’s assessment of identified ADRs). ADRs reporting forms were filled up by the ward pharmacist when an adverse drug reaction (ADR) was detected. Approximately 1–2 pharmacists will be routinely placed in a ward for each shift to monitor ADRs and to assist in other clinical functions during the duration of this study [22].

The date of the clinical or biological diagnosis of the ADR was the start of the ADR. The end of the ADR was the date of normalization of the effect obtained from the ADR reporting form and justified by the date of laboratory examination with normal results or the disappearance of clinical symptoms as reported by doctors and pharmacist. Therefore, if the end date of ADR and the date of the patient’s demise were reported on the same day, the cause of death is considered due to ADRs. The ADRs reportedly lasted from one day to a few weeks [22]. In this study, ADRs were defined in accordance with the Edwards and Aronson classification system [23]. Diagnoses were coded in accordance with International Classification of Disease (10th revision) [24].

### Description of statistical analysis

Patient’s demographic data, physical examination results, comorbid conditions, laboratory tests and medication usage data were compared based on mortality due to ADRs during hospitalization. Continuously distributed variables were analyzed using either T-test or Mann-Whitney U test depending on skewness of data, whereas for categorical variables analysis was performed using the chi-square (x^2^) test. All statistical analysis was performed using IBM SPSS (version 22; SPSS, Inc., Chicago, IL) statistical software. Two-sided *p*-values of less than 0.05 were considered statistically significant.

### Description of study outcome and steps involved in model development

Mortality was fixed as the outcome in the risk model development. Variables with missing values of more than 10% were not considered for analysis. For variables with less than 10% of missing variables, 5 imputations were imputed to the missing data using the multiple imputation technique. Initially, univariate analysis was performed for the selection of variables with *p* < 0.05 that can be included in the subsequent multiple logistic regression analysis. Subsequently, multicollinearity test was performed for all the selected variables to access the presence of any strong correlation between the variables. Mortality risk models were developed by continuously adding patients’ comorbidities, type of medication, laboratory results, total medication use and type of renal replacement therapy. Variables with *p* > 0.10 probability value were excluded from the subsequent analysis. The final model was adjusted with age, sex and estimated GFR. Variables in the final model was presented as odds ratio (OR) with 95% confidence interval (CI).

### Description of model performance

For each risk model, calibration was measured using the Hosmer and Lemeshow goodness of fit test by adding new variables in multiple logistic regression [25]. Hosmer – Square statistics are commonly used to evaluate calibration [19]. Comparison of the overall model effect size for sequential models were done using Nagelkerke R square and *p-*value.

### Description of internal model validation

The final risk model’s internal validity was evaluated using the bootstrapping method. 1000 bootstrap resamples were used to assess the reliability of the coefficients of regression. Standard errors were subsequently used to calculate the 95% bootstrap CI of OR. Conclusion can be made about the population of the data originated using the bootstrapping resampling method [20].

### Development of mortality risk score

Final risk model variables OR were used to calculate the risk scores. The scores were calculated for each patient by an arithmetic sum of points for the present variables. Patients were then categorized according to their risk scores into different cut-off points. Differences in the event rate for increased risk score values for mortality were evaluated using the x^2^ test for trend [26]. Finally, the score’s predictive capacity was assessed using the area under the curve (AUC).

## Results

### Univariate analysis

One thousand and seventy patients’ medical records from multidisciplinary wards were screened during the three-year study period, out of which only 160 medical records fulfill the requirements of the inclusion and exclusion criteria. From the 160 eligible patients, 132 survived and 28 did not survive ADRs during hospitalization. Univariate analysis was performed between these two patient groups (Table 1). Significant difference in survival were observed from variables such as renal function (*p =* 0.041), dyslipidemia (*p* = 0.019), UTI (*p* = 0.005), serum albumin (*p* = < 0.001), serum alkaline phosphatase (*p* = 0.009), serum aspartate aminotransferase (*p* = 0.009), serum CO_2_ (*p* = 0.020), serum C-Reactive protein (*p* = < 0.001), serum lactate dehydrogenase(*p* = < 0.001), low density lipoprotein (*p* = < 0.001) and total medication used during hospitalization (*p* = < 0.001).

**Table 1:** Comparison of Patients Characteristics According to Mortality due to ADRs events

	No. (%) of participants		
Characteristics	Survived (*n* = 132)	Died (*n* = 28)	*p* value
Demographics			
Age			0.888
≤ 49 years	34 (21.3)	7 (4.4)	
50–59 years	32 (20.0)	8 (5.0)	
≥ 60 years	66 (41.3)	13 (8.1)	
Gender			0.643
Male	77 (48.1)	15 (9.4)	
Female	55 (34.4)	13 (8.1)	
Ethnicity			
Malay	44 (27.5)	8 (5.0)	0.626
Chinese	57 (35.6)	11 (6.9)	
Indian	27 (18.1)	9 (5.6)	
Currently or previously smoking	34 (21.3)	7 (4.4)	0.934
Currently or previously consumed alcohol	19 (11.9)	4 (2.5)	0.988
Renal Replacement Therapy			0.750
Haemodialysis	52 (32.5)	9 (5.6)	
Peritoneal dialysis	10 (6.3)	2 (1.3)	
Conservative management	70 (43.8)	17 (10.6)	
Renal Function			0.041
30–59 mL/min/1.73m^2^	46 (28.7)	3 (1.9)	
15–29 mL/min/1.73m^2^	22 (13.8)	7 (4.4)	
< 15 mL/min/1.73m^2^	64 (40.0)	18 (11.3)	
Physical examinations			
Systolic, mean (SD), mm Hg	132 (100)	28 (100)	0.486
Diastolic, median (IQR), mm Hg	132 (100)	28 (100)	0.159
Comorbid conditions			
Diabetes	89 (67.4)	17 (60.7)	0.495
Dyslipidaemia	65 (40.6)	7 (4.4)	0.019
Hypertension	6 (3.8)	22 (13.8)	0.547
UTI	3 (1.9)	4 (2.5)	0.005
Laboratory data			
Serum Albumin, mean (SD), g/L	132 (100)	28 (100)	< 0.001
Serum Alkaline Phosphatase, median (IQR), U/L	131 (99)	28 (100)	0.009
Serum Aspartate Aminotransferase, median (IQR), U/L	94 (71)	22 (79)	0.009
Serum CO2, mean (SD), mmol/L	116 (88)	26 (93)	0.020
Serum C-Reactive Protein, median (IQR), mg/L	62 (47)	23 (82)	< 0.001
Serum Lactate Dehydrogenase, median (IQR), U/L	94 (71)	23 (82)	< 0.001
Serum Low Density Lipoprotein (LDL), mean (SD), mmol/L	62 (50)	6 (21)	< 0.001
VMedications use			
Total medication, median (IQR)	132 (100)	28 (100)	< 0.001

### Model performance

The OR variables for Hosmer and Lemeshow Test and Nagelkerke R square for successive models are shown in Table 2. Model 1 comprising comorbidities heart disease, dyslipidaemia and electrolyte imbalance performed poorly (Hosmer and Lemeshow Test =0.418; Nagelkerke R square = 0.127 and *p* = 0.005). Subsequently, Hosmer and Lemeshow and Nagelkerke R square improved in model 2 with the addition of psychotic agents (Hosmer and Lemeshow Test =0.860; Nagelkerke R square = 0.267 and *p <* 0.001). Continuous improvement was observed with the inclusion of creatinine kinase in the model 3 (Hosmer and Lemeshow Test =0.623; Nagelkerke R square = 0.329 and *p <* 0.001) and total medication use in model 4 (Hosmer and Lemeshow Test =0.899; Nagelkerke R square = 0.393 and *p <* 0.001) and conservative management in model 5 (Hosmer and Lemeshow Test =0.643; Nagelkerke R square = 0.399 and *p <* 0.001). Model 5 have been selected as the best model.

**Table 2:** Odd Ratio and Goodness of Fit for Sequential Models of Mortality Predictions of ADR events

Variable	Models				
	1 Comorbidities	2 Comorbidities + Type of Medication	3 Comorbidities + Type of Medication + Lab Results	4 Comorbidities + Type of Medication + Lab Results + Total Medication Use	5 Comorbidities + Type of Medication + Lab Results + Total Medication Use + Renal Replacement Therapy
^a^ Heart Disease	0.44 (0.18–1.08)	0.44 (0.17–1.15)	0.43 (0.16–1.15)	0.42 (0.15–1.19)	0.46 (0.16–1.32)
Dyslipidaemia	0.32 (0.13–0.84)	0.25 (0.09–0.70)	0.21 (0.07–0.64)	0.24 (0.08–0.73)	0.23 (0.07–0.71)
^b^ Electrolyte Disorder	3.56 (1.19–10.70)	6.36 (1.92–21.08)	5.79 (1.70–19.77)	5.31 (1.49–18.88)	5.72 (1.57–20.89)
^c^ Psychotic Agents		9.42 (3.04–29.12)	8.78 (2.68–28.81)	6.13 (1.77–21.26)	6.02 (1.76–20.64)
Creatinine Kinase ≥171 U/L			6.72 (1.70–26.52)	7.68 (1.75–33.66)	6.81 (1.49–31.20)
≥ 23 No. medications				4.27 (1.49–12.24)	4.66 (1.58–13.79)
Conservative management					1.59 (0.55–4.66)
Hosmer and Lemeshow Test	0.418	0.860	0.623	0.899	0.643
Nagelkerke R Square	0.127	0.267	0.329	0.393	0.399
*p* value	0.005	< 0.001	< 0.001	< 0.001	< 0.001

Abbreviation: GFR, glomerular filtration rate

Data are presented as odd ratios (95% confidence interval) unless otherwise stated

^a^Heart Disease is defined as presence of vascular or/and heart failure aetiology

^b^Electrolyte Disorder is defined as presence of hypokalaemia or hyperkalaemia

^c^Psychrotic Agents is defined as drugs that are classified as psychotropic drugs

^d^SI conversion: To convert Creatinine kinase to μkat/L, multiply by 58.82

### Model validation

Model 5’s logistical regression results with its 1000 sample bootstrap results were tabulated in Table 3. The bootstrapping procedure did not change significant variables as observed.

**Table 3:** Logistic Regression and Bootstrapping Model of Mortality Predictions after ADR events

	Logistic Regression			
Variable	SE	OR (95% CI)	Bootstrap SE	Bootstrap (95% BootCI)
Heart Disease	0.535	0.46 (0.16–1.32)	0.642	(0.37–2.20)
Dyslipidaemia	0.577	0.23 (0.07–0.71)	0.753	(0.42–3.44)
Electrolyte Disorder	0.661	5.72 (1.57–20.89)	0.779	(0.50–3.55)
Psychotic Agents	0.628	6.02 (1.76–20.64)	0.820	(0.41–3.65)
Creatinine Kinase ≥171 U/L	0.777	6.81 (1.49–31.20)	1.842	(0.17–4.46)
≥ 23 No. medications	0.553	4.66 (1.58–13.79)	0.923	(0.49–3.13)
Conservative management	0.547	1.59 (0.55–4.66)	0.694	(0.74–2.05)

Abbreviations: BootCI, bootstrap confidence interval; CI, confidence interval; GFR, glomerular filtration rate; SE, standard error; OR, odd ratio

Data are presented as odd ratios (95% confidence interval) unless otherwise stated

^a^Heart Disease is defined as presence of vascular or/and heart failure aetiology

^b^Electrolyte Disorder is defined as presence of hypokalaemia or hyperkalaemia

^c^Psychrotic Agents is defined as drugs that are classified as psychotropic drugs

^d^SI conversion: To convert Creatinine kinase to μkat/L, multiply by 58.82

### Mortality risk score

Model 5 variables were assigned with scores based on their OR value, such as heart disease (OR: 0.463): 1; dyslipidemia (OR: 0.230): 0; electrolyte disorder (OR: 5.717): 6; psychotic agents (OR:6.021): 6; creatinine kinase ≥171 U/L (OR:6.811): 7; ≥ 23 number of medications (OR:4.663):5 and conservative management (OR: 1.595): 2 (Table 4). Creatinine kinase was the most strongly associated variable with mortality after ADRs during hospitalization and followed by psychotic agents, electrolyte disorder and total number of medications with median value of ≥23. The conservative management, heart disease and dyslipidaemia have moderate mortality effect on these CKD patients.

**Table 4:** Regression Coefficient and Score of Each Variable Included in Predictive Model

Variable	OR (95% CI)	Score
Comorbidities		
Heart Disease	0.46 (0.16–1.32)	**+ 1**
Dyslipidaemia	0.23 (0.07–0.71)	**+ 0**
Electrolyte Disorder	5.72 (1.57–20.89)	**+ 6**
Type of Medication		
Psychotic Agents	6.02 (1.76–20.64)	**+ 6**
Lab Results		
Creatinine Kinase ≥171 U/L	6.81 (1.49–31.20)	**+ 7**
Total Medication Use		
≥ 23 No. medications	4.66 (1.58–13.79)	**+ 5**
Renal Replacement Therapy		
VConservative management	1.59 (0.55–4.66)	**+ 2**

The score was calculated for each patient by assigning points and adding these points for each variable present. For example, an end stage renal disease (ESRD) patient undergoing conservative management, with presence of comorbidities as heart disease, dyslipidaemia and electrolyte imbalance, consuming psychotic drugs, with creatinine kinase lab value of ≥171 U/L and consuming ≥23 of total number of medications receives a score of (2 + 1 + 0 + 6 + 6 + 7 + 5) = 27.

The range of the score in this study is 0 (minimum) to 21 (maximum); the calculated mean (SD) was 7.03 (SE ±0/43); the calculated median was 7.00; and AUC was 0.789 (95% CI, 0.700–0.878). Incidence of mortality due to ADRs during hospitalization among stages 3–5 CKD patients across various score categories were tabulated in Table 5. From this table, the observed mortality per score category can be calculated directly when reading the score horizontally. For example, of 50 patients with a score of ≥10, 71.4% (*n* = 20) did not survive ADRs, while 22.7% (*n* = 30) survived ADRs during hospitalization. Only 3.6% (*n* = 1) from the total of 28 patients with score of 0–1 did not survive ADRs during hospitalization. Reading Table 5 vertically provides the estimates of sensitivity and specificity at different cut points. For instance, 50 patients received a score of ≥10. Of these, 20 patients died after ADRs event during hospitalization, correctly predicting 71.4% of all mortality pertaining to ADRs (sensitivity). Since 30 (22.7%) of all patients survived after ADRs during hospitalization, the cut-point specificity was 100–22.7 = 77.3%.

**Table 5:** Distribution of Patients According Risk Score Derived From Model 5

Risk Score	Total^a^	No. patients died after an ADR, n (%)	No. patients survived after an ADR, n (%)
0–1	28	1 (3.6)	27 (20.5)
2–3	32	1 (3.6)	31 (23.5)
4–5	8	2 (7.1)	6 (4.5)
6–7	26	1 (3.6)	25 (18.9)
8–9	16	3 (10.7)	13 (9.8)
≥10	50	20 (71.4)	30 (22.7)
Total	160	28	132

Abbreviations: ADR, adverse drug reaction

^a^Total number of patients per score category

## Discussions

Mortality risk score model was developed in this study with modest effect (R square = 0.399) and with AUC = 0.789 (95% CI, 0.700–0.878). The developed mortality risk score uses routinely available clinical data that can be combined into the clinical practice as an identification tool for CKD patients whom are at high risk mortality due to ADRs. In Hospital Pulau Pinang yearly about 13.5% of hospitalized CKD patients stages 3 to 5 experienced ADRs. In addition, approximately 88% of ADRs in this study were possibly preventable. Severity assessment performed indicated that nearly 27% of ADRs scored mild and nearly 60% of ADRs scored at level 3 or below on the Hartwig scale [22].

ADRs have been listed in many countries as among the leading causes of mortality. This called for efforts to raise patient awareness and to further motivate healthcare professionals in healthcare facilities to continuously monitor and report ADRs in order to minimize its risk [27–29]. Although the consequences of ADRs on patients were reported in various studies, the incidence and patterns of ADRs data is very minimally reported. Factors contributing to ADRs include an increase in the number of drugs sold, drug type, an increase in the aging population, pregnancy, gender, hormonal and immunological factors, differences in pharmacokinetics, patterns of drug usage, disease state, genetics, ethnicity, polypharmacy and urbanization [30–32].

In multivariable analysis, it is recommended to report the Nagelkerke R square values when measuring the strength of the predictor variables, as it describes the variance between model prediction and outcome measurement [18]. Tangiisuran et al. (2014) reported low R square value of 0.16, which corresponds to the predictive capacity of their ADR risk score. They argued that the model provides more ADR risk information despite the small effect size than without predictors [33].

Both vascular disease and dyslipidaemia remained important predictors in this study when included in the regression model simultaneously. Tangri et al. (2013) reported that absolute rates of cardiovascular events were higher among CKD patients compared to the general population [21]. This were attributable to the high prevalence of hypertension, dyslipidaemia, hyperuricemia, abnormal glucose metabolism, obesity, high incidence of cardiovascular disease (CVD), systemic inflammation and oxidative stress among CKD patients. CKD associated cardiovascular alterations were influenced by rapid ageing process associated with shortening of telomere length [34]. However, mechanisms underlying the accelerated vascular and cardiac calcification state that were profound among CKD and ESRD were not completely understood [35]. Moreover, during early stages of CKD, atherosclerotic processes such as invasion of the macrophage, plaque formation and thickening of the arterial wall were the major events that happen in the human body. However, during later stages of CKD, vascular wall degeneration occurs, influenced by inflammatory factors and media calcification. Therefore, involvement of various factors results in discrete changes, in the risk factor profile, that inversely contribute to outcomes during CKD progression [36].

Dyslipidaemia which is a common comorbidity among CKD patients is an important risk factor for the development of CVD as it contributes to the alteration of lipoprotein metabolism and is associated with GFR decline [37, 38]. Among CKD patients, abnormal lipid metabolism and changes in lipid particles related to uremic toxin promoting atherogenesis were common [39]. Post-translational alterations of lipid particles which were due to CKD causes proinflammatory effects and endothelial dysfunction [40]. Monitoring of hyperlipidaemia by modulating the transport of lipids, suppressing systemic inflammation, modulating the intestinal microbiota or vascular calcification and interference with heart fibrosis can help to a [35].

Creatinine kinase and psychotic agents were most strongly associated with mortality in this study. Phosphorylation of creatine into phosphocreatinine are catalysed by serum creatine kinase (sCK) or creatine phosphokinase (CPK) which is a cytosolic and mitochondrial enzyme. Subsequently, phosphocreatine aids in adenosine diphosphate (ADP) conversion to adenosine triphosphate (ATP) [41]. Muscle mass deterioration is a predictor for mortality in CKD patients in whom the serum creatinine is found in high quantities [41, 42].

The Finnish group reported that the antipsychotic and antidepressants were common drugs involved in mortality [43]. Moreover, Tsai et al. (2012) reported that consumption of anti-depressive agents such as psychotic drugs by ESRD patients resulted in significantly higher mortality rate compared to their counterparts [44]. Anti-depressant medications are highly protein-bound, metabolized hepatically and are not significantly removed by dialysis [45, 46]. Psychotropic medications were associated with increased risk of multiple physical diseases such as obesity, dyslipidaemia, diabetes mellitus, thyroid disorders, hyponatremia, cardiovascular, respiratory, gastrointestinal, haematological, musculoskeletal and renal diseases, movement and seizure disorders [47]. It also been reported that the metabolic abnormalities accumulate rapidly after the treatment has been started [48, 49]. Furthermore, poor compliance with treatment, higher peritonitis rates in chronic peritoneal dialysis, lack of intention to change their lifestyle and abnormalities in the functioning of the immune system ultimately results in mortality for ESRD patients [50, 51]. Although mental illnesses such as depression and anxiety were common among CKD patients, very limited studies have been performed [52].

One of the strong predictors of mortality from this study is the presence of electrolyte imbalances condition. Hypokalaemia and hyperkalaemia were typical disorders of the electrolyte due to changes in potassium intake, altered excretion or transcellular shifts. In ESRD patients, the mortality rate attributed to hypokalaemia was 2 to 5% and occurred in up to 21% among hospitalized patient population. Diuretic use and gastrointestinal losses cause hypokalaemia. On the other hand, hyperkalaemia was associated with 17% higher mortality rate. The risk of hyperkalaemia is increased by the presence of comorbid conditions such as chronic kidney disease, heart failure, diabetes and liver disease in combination with the use of certain medications [53–55].

Additionally, the total number of medications, conservatively managed ESRD and the presence of comorbidities such as heart disease and dyslipidaemia were important predictors of mortality from this study. Goldberg and colleagues reported that taking medication simultaneously increases the risk of ADRs. The risk of ADRs was increased to 13% with two concurrent medications, to 38% with four concurrent medications and to 82% with seven or more medications [56]. Moreover, prescribing multiple drugs also increases the risk of drug interactions [57].

In this study, patients who were not dependent on dialysis had twice the risk of mortality due to ADRs compared to dialysis patients. In patients with CKD who managed their disease conservatively, the risk of cardiovascular events is as high as in people with confirmed coronary artery disease. The risk is amplified with increased insulin resistance, increased blood pressure, calcification of the vascular system, inflammation and deterioration of protein energy [58–61]. It was reported that 57% of ESRD patients did not receive replacement therapy based on the Indian CKD registry reports [1], which increases their mortality risk.

The treatment with hemodialysis leads to a reduced risk of death compared to conservative treatment. Hemodialysis may, however, deteriorate further in older patients’ functional status and quality of life. This may due to, very frequent attendance of dialysis session causing very little time for other activities, including nutritional intake. In addition, older patients with hemodialysis experiences frequent burden symptoms such as pain, tiredness, pruritus and constipation [62].

Numerous median survival reports were available for the conservatively managed cohort ranging from 8.9 to 22 months [63–65]. However, comparing survival in conservatively managed patients between different cohorts compared to patients receiving renal replacement therapy is difficult because there is no clear starting point for analysis and therefore leads to lead time bias [65]. The time to initiate renal replacement therapy in the elderly remains intangible and international societies’ recommendations have changed over the past decade. Time to event analysis showed better survival in conservatively managed patients who survived for more than two months [65–67]. Conversely, Shum et al. (2014) reported that peritoneal dialysis may be an appropriate treatment option for elderly ESRD patients [68].

Accurate mortality risk predictions among stage 3 to 5 CKD patients are important for providing evidence about a wide range of interventions that often results in impacts on clinical practice and research that were associated with benefits and harms. Risk prediction models were generally based on demographics and laboratory variables that are typically available in clinical practice in the real world. However, developed risk models tend to be population-specific and most of the CKD prediction models available were based on European population [69]. A systematic review of the CKD risk prediction tools found that the discriminative performance of these models is considered acceptable to good in the population in which they were developed and modest to acceptable if tested in a different population [70, 71].

Risk models are also useful in personalizing patient management with different risk levels to reduce mortality outcomes and further improve the cost-effectiveness of CKD therapy [72]. In addition, regularly measured laboratory variables would facilitate the timely calculation of risk from electronic medical records or laboratory information systems [21]. Conclusively, this study incorporated laboratory data of the CKD patients in the final mortality prediction model. Hence, this risk prediction model for mortality is highly relevant and applicable for routine and bed-side use in clinical practice.

## Strengths and limitations

The strength of this study lies in the identification of ADRs by using a 3-step identification method. In addition, the study population was from multiple wards representing the clinical specialties commonly found in most acute hospitals and our study population age distribution was comparable to the figures for all hospitalized patients from other literatures. It is therefore likely to produce highly reliable results that are more representative of the practice of the real world. Finally, the mortality risk score is practicable as all the variables included can be obtained regularly in the hospital setting and can thus be translated into use at patients’ bedside.

The limitation of this study is that it was conducted in one hospital and it is likely that there will be variations between different hospitals due to differences in local population characteristics and hospital specialties. The low study power and modest performance of the developed model is attributable to small number of eligible patients’ medical records from the total number screened for the purpose of this study. Furthermore, CKD patients often have other comorbid conditions requiring careful evaluation of the causal relationship between ADRs and mortality. Moreover, this risk score is not externally validated which could limit the generalizability of the results and survival risks for specific stages may be overestimated because they were derived using only one GFR measurement.

## Conclusion

Mortality prediction model was developed in this study for stages 3 to 5 CKD patients who experienced ADRs during hospitalization. This tool is clinically useful and effective in preliminary identification of potential CKD patients whom are at high risk of ADR-related mortality during hospitalization using routinely performed clinical data.

## Recommendation

External validation studies can be performed to enhance the generalizability of the developed mortality risk prediction model.

## Data Availability

The datasets generated and analysed during the current study are not publicly available due to data confidentiality policy as dictated in the study approval letter by the Medical Research & Ethics Committee (MREC), Ministry of Health Malaysia (MOH) (Ref no: (5) KKM/NIHSEC/P16–25) but are available from the corresponding author on reasonable request.

## Abbreviations

ADRs: Adverse drug reactions
AUC: Area under the curve
CKD: Chronic kidney disease
eGFR: estimated glomerulus filtration
GFR: Glomerular filtration rate
OR: Odd ratios
SD: Standard deviation

## References

[1] Jha V (2009). Current status of chronic kidney disease care in Southeast Asia. Semin Nephrol.

[2] Hooi LS, Ong LM, Ahmad G (2013). A population-based study measuring the prevalence of chronic kidney disease among adults in West Malaysia. Kidney Int.

[3] Thomas B, Matsushita K, Abate KH (2017). Global cardiovascular and renal outcomes of reduced GFR. Journal of the American Society of Nephrology : JASN.

[4] GBD. Global (2016). Regional, and national life expectancy, all-cause mortality, and cause-specific mortality for 249 causes of death, 1980-2015: a systematic analysis for the global burden of disease study 2015. Lancet (London, England)..

[5] Kerr PG, Tran HTB, Ha Phan HA (2018). Nephrology in the Oceania-South East Asia region: perspectives and challenges. Kidney Int.

[6] WHO (2012). Projections of mortality and causes of death, 2015 and 2030.

[7] WHO. Safety of medicines a guide to detecting and reporting adverse drug reactions : why health professionals need to take action. Geneva. p. Switzerland2002.

[8] WHO. World Health Statistics 2010 (2010, accessed 16/2/2017).

[9] Rashed AN, Wong IC, Cranswick N, Tomlin S, Rascher W, Neubert A (2012). Risk factors associated with adverse drug reactions in hospitalised children: international multicentre study. Eur J Clin Pharmacol.

[10] Chan SL, Ang X, Sani LL (2016). Prevalence and characteristics of adverse drug reactions at admission to hospital: a prospective observational study. Br J Clin Pharmacol.

[11] Rosli R, Ming LC, Abd Aziz N, Manan MM (2016). A retrospective analysis of spontaneous adverse drug reactions reports relating to Paediatric patients. PLoS One.

[12] MADRAC. Malaysian guidelines for the reporting and monitoring of ADR: (2002).

[13] MADRAC. Annual Report 2015 National Centre for Adverse Drug Reactions Monitoring, National Pharmaceutical Control Bureau (NPCB): (2015).

[14] Rosli R, Dali AF, Aziz NA, Ming LC, Manan MM (2017). Reported adverse drug reactions in infants: a Nationwide analysis in Malaysia. Front Pharmacol.

[15] Pirmohamed M, Breckenridge AM, Kitteringham NR, Park BK (1998). Adverse drug reactions. BMJ (Clinical research ed).

[16] Ulrich RG (2007). Idiosyncratic toxicity: a convergence of risk factors. Annu Rev Med.

[17] Iasella Carlo J., Johnson Heather J., Dunn Michael A. (2017). Adverse Drug Reactions. Clinics in Liver Disease.

[18] Falconer N, Barras M, Cottrell N (2018). Systematic review of predictive risk models for adverse drug events in hospitalized patients. Br J Clin Pharmacol.

[19] Rigatto Claudio, Sood Manish M., Tangri Navdeep (2012). Risk prediction in chronic kidney disease. Current Opinion in Nephrology and Hypertension.

[20] Steyerberg E.W. (2009). Clinical Prediction Models.

[21] Tangri N, Kitsios GD, Inker LA (2013). Risk prediction models for patients with chronic kidney disease: a systematic review. Ann Intern Med.

[22] Danial M, Hassali MA, Ong LM, Khan AH (2018). Survivability of hospitalized chronic kidney disease (CKD) patients with moderate to severe estimated glomerular filtration rate (eGFR) after experiencing adverse drug reactions (ADRs) in a public healthcare center: a retrospective 3 year study. BMC Pharmacol Toxicol.

[23] Edwards IR, Aronson JK (2000). Adverse drug reactions: definitions, diagnosis, and management. Lancet (London, England).

[24] WHO. International statistical classification of diseases and related Health problems 10th revision. Geneva, Switzerland2016.

[25] Lemeshow S, Hosmer DW (1982). A review of goodness of fit statistics for use in the development of logistic regression models. Am J Epidemiol.

[26] Antman EM, Cohen M, Bernink PJ (2000). The TIMI risk score for unstable angina/non-ST elevation MI: a method for prognostication and therapeutic decision making. Jama..

[27] Hepler C. Understanding adverse drug therapy outcomes. Preventing medication errors and improving drug therapy outcomes: a management systems approach. 2003.

[28] Knudsen P, Herborg H, Mortensen A, Knudsen M, Hellebek A (2007). Preventing medication errors in community pharmacy: frequency and seriousness of medication errors. Quality and safety in Health care.

[29] Schnipper JL, Kirwin JL, Cotugno MC (2006). Role of pharmacist counseling in preventing adverse drug events after hospitalization. Arch Intern Med.

[30] Hinson Jack A., Roberts Dean W., James Laura P. (2009). Mechanisms of Acetaminophen-Induced Liver Necrosis. Handbook of Experimental Pharmacology.

[31] Shepherd G, Mohorn P, Yacoub K, May DW (2012). Adverse drug reaction deaths reported in United States vital statistics, 1999-2006. Ann Pharmacother.

[32] Tan Y, Hu Y, Liu X, Yin Z, Chen XW and Liu M. Improving drug safety: from adverse drug reaction knowledge discovery to clinical implementation. Methods *(*San Diego, Calif*)*. 2016. 10.1016/j.ymeth.2016.07.023 27485605

[33] Tangiisuran B, Scutt G, Stevenson J (2014). Development and validation of a risk model for predicting adverse drug reactions in older people during hospital stay: Brighton adverse drug reactions risk (BADRI) model. PLoS One.

[34] Raschenberger J, Kollerits B, Titze S (2015). Association of relative telomere length with cardiovascular disease in a large chronic kidney disease cohort: the GCKD study. Atherosclerosis..

[35] Romagnani P, Remuzzi G, Glassock R (2017). Chronic kidney disease. Nature Reviews Disease Primers.

[36] Grabner A, Amaral AP, Schramm K (2015). Activation of cardiac fibroblast growth factor receptor 4 causes left ventricular hypertrophy. Cell Metab.

[37] Mikolasevic I, Zutelija M, Mavrinac V, Orlic L (2017). Dyslipidemia in patients with chronic kidney disease: etiology and management. Int J Nephrol Renov Dis.

[38] Cases Aleix, Coll Elisabet (2005). Dyslipidemia and the progression of renal disease in chronic renal failure patients. Kidney International.

[39] Vaziri ND (2006). Dyslipidemia of chronic renal failure: the nature, mechanisms, and potential consequences. American journal of physiology Renal physiology.

[40] Speer T, Rohrer L, Blyszczuk P (2013). Abnormal high-density lipoprotein induces endothelial dysfunction via activation of toll-like receptor-2. Immunity..

[41] Flahault A, Metzger M, Chasse JF (2016). Low serum Creatine kinase level predicts mortality in patients with a chronic kidney disease. PLoS One.

[42] Macdonald JH, Marcora SM, Kumwenda MJ (2006). The relationship between estimated glomerular filtration rate, demographic and anthropometric variables is mediated by muscle mass in non-diabetic patients with chronic kidney disease. Nephrol Dial Transplant.

[43] Dormann H, Criegee-Rieck M, Neubert A (2003). Lack of awareness of community-acquired adverse drug reactions upon hospital admission : dimensions and consequences of a dilemma. Drug Saf.

[44] Tsai C-J, Loh E-W, Lin C-H, Yu T-M, Chan C-H, Lan T-H (2012). Correlation of antidepressive agents and the mortality of end-stage renal disease. Nephrology..

[45] Hedayati SS, Finkelstein FO (2009). Epidemiology, diagnosis, and management of depression in patients with CKD. Am J Kidney Dis.

[46] Bautovich A, Katz I, Smith M, Loo CK, Harvey SB (2014). Depression and chronic kidney disease: a review for clinicians. Aust N Z J Psychiatry.

[47] Correll CU, Detraux J, De Lepeleire J, De Hert M (2015). Effects of antipsychotics, antidepressants and mood stabilizers on risk for physical diseases in people with schizophrenia, depression and bipolar disorder. World psychiatry : official journal of the World Psychiatric Association (WPA).

[48] Chesney E, Goodwin GM, Fazel S (2014). Risks of all-cause and suicide mortality in mental disorders: a meta-review. World psychiatry : official journal of the World Psychiatric Association (WPA).

[49] De Hert M, Detraux J, van Winkel R, Yu W, Correll CU (2011). Metabolic and cardiovascular adverse effects associated with antipsychotic drugs. Nat Rev Endocrinol.

[50] Kimmel PL, Cukor D, Cohen SD, Peterson RA (2007). Depression in end-stage renal disease patients: a critical review. Adv Chronic Kidney Dis.

[51] Kimura H, Ozaki N (2006). Diagnosis and treatment of depression in dialysis patients. Therapeutic apheresis and dialysis : official peer-reviewed journal of the International Society for Apheresis, the Japanese Society for Apheresis, the Japanese Society for Dialysis Therapy.

[52] Pereira BDS, Fernandes NDS, de Melo NP, Abrita R, Grincenkov F, Fernandes N (2017). Beyond quality of life: a cross sectional study on the mental health of patients with chronic kidney disease undergoing dialysis and their caregivers. Health Qual Life Outcomes.

[53] Schulman Michael, Narins Robert G. (1990). Hypokalemia and cardiovascular disease. The American Journal of Cardiology.

[54] Viera AJ, Wouk N (2015). Potassium Disorders: Hypokalemia and Hyperkalemia. Am Fam Physician.

[55] Korgaonkar S, Tilea A, Gillespie BW (2010). Serum potassium and outcomes in CKD: insights from the RRI-CKD cohort study. Clin J Am Soc Nephrol.

[56] Goldberg RM, Mabee J, Chan L, Wong S (1996). Drug-drug and drug-disease interactions in the ED: analysis of a high-risk population. Am J Emerg Med.

[57] Juurlink DN, Mamdani M, Kopp A, Laupacis A, Redelmeier DA (2003). Drug-drug interactions among elderly patients hospitalized for drug toxicity. Jama..

[58] De Cosmo S, Menzaghi C, Prudente S, Trischitta V (2013). Role of insulin resistance in kidney dysfunction: insights into the mechanism and epidemiological evidence. Nephrol Dial Transplant.

[59] Cozzolino M, Ketteler M, Zehnder D (2010). The vitamin D system: a crosstalk between the heart and kidney. Eur J Heart Fail.

[60] Vervloet M, Cozzolino M (2017). Vascular calcification in chronic kidney disease: different bricks in the wall?. Kidney Int.

[61] Carrero JJ, Stenvinkel P, Cuppari L (2013). Etiology of the protein-energy wasting syndrome in chronic kidney disease: a consensus statement from the International Society of Renal Nutrition and Metabolism (ISRNM). J Ren Nutr.

[62] Burns A, Davenport A (2010). Maximum conservative management for patients with chronic kidney disease stage 5. Hemodialysis international International Symposium on Home Hemodialysis.

[63] Murtagh FE, Marsh JE, Donohoe P, Ekbal NJ, Sheerin NS, Harris FE (2007). Dialysis or not? A comparative survival study of patients over 75 years with chronic kidney disease stage 5. Nephrol Dial Transplant.

[64] Chandna SM, Da Silva-Gane M, Marshall C, Warwicker P, Greenwood RN, Farrington K (2011). Survival of elderly patients with stage 5 CKD: comparison of conservative management and renal replacement therapy. Nephrol Dial Transplant.

[65] Reindl-Schwaighofer R, Kainz A, Kammer M, Dumfarth A, Oberbauer R (2017). Survival analysis of conservative vs. dialysis treatment of elderly patients with CKD stage 5. PLoS One.

[66] Moss AH (2010). Revised Dialysis clinical practice guideline promotes more informed decision-making. Clin J Am Soc Nephrol.

[67] Johnson DW, Wong MG, Cooper BA (2012). Effect of timing of dialysis commencement on clinical outcomes of patients with planned initiation of peritoneal dialysis in the IDEAL trial. Perit Dial Int.

[68] Shum CK, Tam KF, Chak WL, Chan TC, Mak YF, Chau KF (2014). Outcomes in older adults with stage 5 chronic kidney disease: comparison of peritoneal dialysis and conservative management. J Gerontol A Biol Sci Med Sci.

[69] Echouffo-Tcheugui JB, Kengne AP (2012). Risk models to predict chronic kidney disease and its progression: a systematic review. PLoS Med.

[70] Moons KG, Kengne AP, Grobbee DE (2012). Risk prediction models: II. External validation, model updating, and impact assessment. Heart.

[71] Moons KGM, de Groot JAH, Bouwmeester W (2014). Critical appraisal and data extraction for systematic reviews of prediction modelling studies: the CHARMS checklist. PLoS Med.

[72] Saheb Sharif-Askari F, Syed Sulaiman SA, Saheb Sharif-Askari N, Al Sayed Hussain A (2014). Development of an adverse drug reaction risk assessment score among hospitalized patients with chronic kidney disease. PLoS One.

